# Occurrence and Molecular Phylogeny of Honey Bee Viruses in Vespids

**DOI:** 10.3390/v12010006

**Published:** 2019-12-19

**Authors:** Sa Yang, Philippe Gayral, Hongxia Zhao, Yaojun Wu, Xuejian Jiang, Yanyan Wu, Diane Bigot, Xinling Wang, Dahe Yang, Elisabeth A. Herniou, Shuai Deng, Fei Li, Qingyun Diao, Eric Darrouzet, Chunsheng Hou

**Affiliations:** 1 Institute of Apicultural Research, Chinese Academy of Agricultural Sciences, Beijing 100093, China; sayang1994@163.com (S.Y.); wuyanyan@caas.cn (Y.W.); wangxinlingjiayou@126.com (X.W.); yangdahe@126.com (D.Y.); shdeng11@163.com (S.D.); dqyun1@126.com (Q.D.); 2 Key Laboratory of Pollinating Insect Biology, Ministry of Agriculture, Beijing 100093, China; 3 Institut de Recherche sur la Biologie de l’Insecte, UMR 7261, CNRS—Université de Tours, F-37200 Tours, France; philippe.gayral@univ-tours.fr (P.G.); bigot.diane@gmail.com (D.B.); elisabeth.herniou@univ-tours.fr (E.A.H.); 4 Guangdong Key Laboratory of Animal Conservation and Resource Utilization, Guangdong Public Laboratory of Wild Animal Conservation and Utilization, Guangdong Institute of Applied Biological Resources, Guangzhou 510260, China; hxzh110@126.com; 5 Institute of Forestry Protection, Guangxi Zhuang Autonomous Region Forestry Research Institute, Nanning 530002, China

**Keywords:** honeybee viruses, hornets, AmFV, IAPV, DWV

## Abstract

Since the discovery that honey bee viruses play a role in colony decline, researchers have made major breakthroughs in understanding viral pathology and infection processes in honey bees. Work on virus transmission patterns and virus vectors, such as the mite *Varroa destructor*, has prompted intense efforts to manage honey bee health. However, little is known about the occurrence of honey bee viruses in bee predators, such as vespids. In this study, we characterized the occurrence of 11 honey bee viruses in five vespid species and one wasp from four provinces in China and two vespid species from four locations in France. The results showed that all the species from China carried certain honey bee viruses, notably *Apis mellifera filamentous virus* (AmFV), *Deformed wing virus* (DWV), and *Israeli acute paralysis virus* (IAPV); furthermore, in some vespid colonies, more than three different viruses were identified. In France, DWV was the most common virus; *Sacbrood virus* (SBV) and *Black queen cell virus* (BQCV) were observed in one and two samples, respectively. Phylogenetic analyses of IAPV and BQCV sequences indicated that most of the IAPV sequences belonged to a single group, while the BQCV sequences belonged to several groups. Additionally, our study is the first to detect *Lake Sinai virus* (LSV) in a hornet from China. Our findings can guide further research into the origin and transmission of honey bee viruses in Vespidae, a taxon of ecological, and potentially epidemiological, relevance.

## 1. Introduction

Honey bees (*Apis mellifera*) make vital contributions to plant diversity and agricultural ecosystems [1], but they are also attacked by a variety of pathogens, including viruses, bacteria, and fungi [2]. Researchers have identified more than 20 honey bee viruses that impact bee health and survival [3]. Among them are eight common viruses thought to pose a lethal threat: *Israeli acute paralysis virus* (IAPV), *Deformed wing virus* (DWV), *Black queen cell virus* (BQCV), *Sacbrood virus* (SBV), *Chronic bee paralysis virus* (CBPV), *Acute bee paralysis virus* (ABPV), *Kashmir bee virus* (KBV), and *Kakugo virus* (DWV-A/KV) [2]. In some cases, a vector influences infection and transmission patterns. For example, the parasitic mite *Varroa destructor* boosts viral titers and expands the transmission range of DWV [4] and IAPV [5]. 

Virus transmission dynamics could also be affected by honey bee predators, a subject about which little is currently known. Hornets, for example, are major bee predators, and the yellow-legged hornet (*Vespa velutina nigrithorax*)—an invasive species in Europe [6]—has a major impact on honey bee populations. At present, public authorities and the beekeeping industry are deeply concerned about the rapid spread of *V. velutina* and its dramatic impact on honey bee survival in Europe [7], South Korea [8,9], and Japan [10]. The yellow-legged hornet was first observed in France in 2005 [11,12]. It quickly spread throughout the country and then moved into other parts of Europe [6,13,14,15]. The species is native to China, where it has also recently begun to significantly threaten honey bee survival [16], especially in the autumn and winter, when pollinating insects face a lack of food resources.

Apiculture is a major industry in China. Beekeepers manage more than 9 million hives and supply enough honey for the country’s consumers. However, the European honey bee (*A. mellifera*) lacks effective defensives against predators like the yellow-legged hornet. Beekeepers in Europe have reported that *V. velutina* preys on honey bee colonies—impacting populations [17]—but quantitative data of this type remain scarce for China (Figure S1). Since viruses can move from primary hosts to new hosts, infected hornets could influence the host dynamics of honey bee viruses. Hornets hunt across broad areas for bees, which means that hornets may come in contact with and carry viruses from multiple honey bee colonies. Several studies have shown that honey bee viruses occur in the yellow-legged hornet [18,19,20], but little research has been dedicated to exploring virus diversity. This study aims to provide a better foundation for understanding honey bee virus dynamics in vespids. 

Vespids of various species were collected from four provinces in China and four locations in France to (1) characterize the occurrence of major honey bee viruses in vespids and (2) explore the phylogenetic relationships among virus sequences from vespids and honey bees. 

## 2. Materials and Methods 

### 2.1. Samples

**Figure 1:**
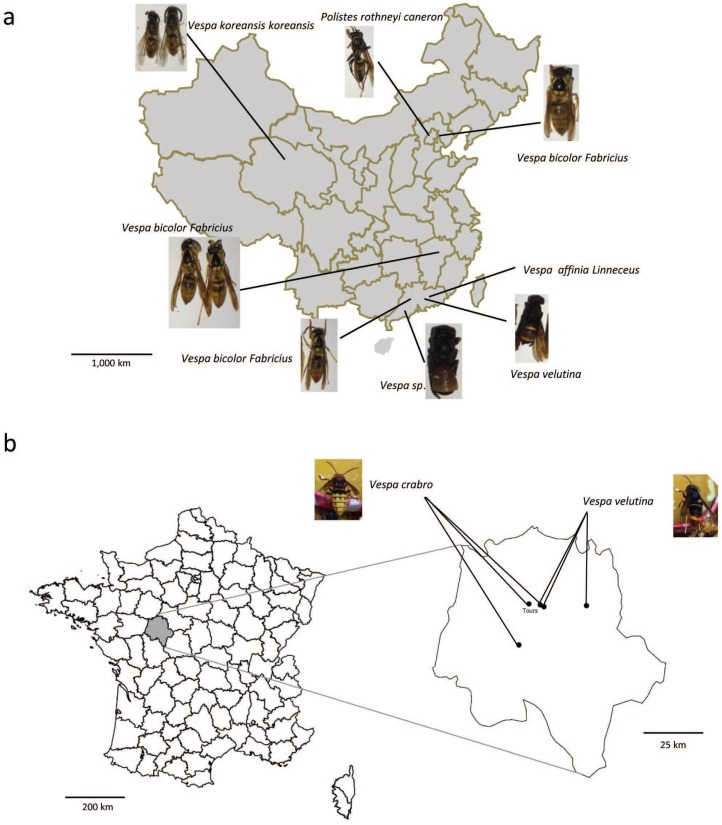
Sampling locations in (**a**) China and (**b**) France. Sampled species are indicated by names and photos.

Vespids and wasps were sampled in 2016 and 2017. In China, 12 colonies were collected in four provinces, including five colonies of *Vespa bicolor*, two colonies of *Polistes rotheyi*, two colonies of *Vespa velutina*, one colony of *Vespa koreansis*, one colony of *Vespa affinia*, one colony of *Vespa sp*. (Figure 1a). For each colony, fifty workers and pupa (if present) were randomly selected and tested for the presence of honey bee viruses. In France, 14 vespids colonies were collected from four locations near the city of Tours, which is in the administrative department of Indre-et-Loire (Figure 1b). The vespids were sampled live, directly transported to the laboratory in a small iron wire cage, quickly freeze killed at −80 °C, and kept frozen until analyses could take place. None of the vespids displayed symptoms of infection.

### 2.2. Identification of Vespid Species

We identified the vespid species collected in China using a DNA barcoding approach. Total genomic DNA was extracted from one individual per colony using the Genomic DNA Kit (CW Biotech, Beijing, China). A universal primer set (LCO-1490 and HCO-2198) was used to amplify a 709 bp fragment of cytochrome c oxidase subunit I (CO I) via polymerase chain reaction (PCR) [21]. The same CO I primers were used to carry out PCR on cDNA to verify that RNA from each colony was of good quality (Figure S2). The PCR conditions for amplifying the CO I gene were as follows: an initial denaturation step at 95 °C for 5 min; 40 cycles of 94 °C for 40 s, 54 °C for 40 s, and 72 °C for 1 min; and a final extension step at 72 °C for 10 min. PCR products were checked using electrophoresis (1.2% agarose gel). Successfully amplified PCR products were purified using a DNA Purification Kit (CW Biotech, Beijing, China); they were then Sanger sequenced employing primers described by Yue et al. [21] (Sango Biotech, Shanghai, China). The sequences were then assembled using Vector NTI Advance software and identified via a BLAST search in GenBank. Vespid species and castes were also identified using morphological characters (as described by Matsuura [22]), and these identifications were confirmed by an expert (Li Yun, Institute of Apicultural Research, CAAS). French vespid species were identified morphologically by a different expert (ED).

### 2.3. Extraction of DNA and RNA for Virus Identification

For the Chinese samples, DNA and RNA were extracted from a pool of approximately 20 workers or pupae per colony; queens were also used, if present. The samples were prepared using cold grinding (liquid nitrogen and a mortar/pestle). Total RNA was extracted using a TRIzol Kit (Invitrogen, Carlsbad, CA, USA) and TissuePrep homogenizer (Gering Scientific Instruments, Beijing, China) in accordance with the manufacturers’ instructions. The resulting sample was then eluted in 20 µL of sterile water and stored at −80 °C. The quantity and purity of the RNA were measured using a Nanodrop spectrophotometer (Thermo Scientific, Beijing, China). We synthesized cDNA from 4 µg of the total RNA using M-MLV reverse transcriptase (Promega, Madison, WI, USA) and the oligo dT primer in accordance with the manufacturer’s instructions. Total DNA was extracted using a Wizard Genomic DNA Purification Kit (Promega, Madison, WI, USA) and TissuePrep homogenizer (Gering Scientific Instruments, Beijing, China) following the manufacturers’ instructions. The resulting sample was then eluted into 20 μL of nuclease-free water and stored at −20 °C.

For the French samples, RNA extraction was performed as previously described [23]. Briefly, the head, abdomen, and thorax of individual vespids were placed in three separate tubes with a 5 mm stainless steel bead. The tissues were then mechanically homogenized for 90 sec (3×) at 30 Hz using a TissueLyser II (Qiagen, Courtaboeuf, France). RA1 lysis buffer (NucleoSpin RNA Isolation Kit, Qiagen, Courtaboeuf, France) was then added in accordance with the manufacturer’s instructions; the amount depended on the sample mass. The lysates of the three body sections were then pooled and centrifuged for 3 min at 14,000× *g*. Next, total RNA extraction was performed using 350 µL of the supernatant and a NucleoSpin RNA Isolation Kit (Macherey-Nagel, Bethlehem, PA, USA) in accordance with the manufacturer’s instructions. The resulting sample was eluted into 40 µL of RNAse free water; this process was repeated three times with the same elute to increase final RNA concentration. RNA quality was visually controlled via electrophoresis (1% agarose gel), and RNA quantity was measured using a Qubit fluorometer. Reverse transcription was performed using a RevertAid First Strand cDNA Synthesis Kit (Life Technologies, Carlsbad, CA, USA) and random hexamer primers in accordance with the manufacturer’s instructions; in most cases, 1 µg of total RNA was employed.

### 2.4. Identification of Honey Bee Viruses via PCR

The primer sequences are provided in Table S1. We used PCR to test the Chinese samples for the presence of 10 RNA viruses (IAPV, SBV, DWV-A, DWV-A/KV, CBPV, ABPV, BQCV, KBV, Deformed wing virus-B, Chinese Sacbrood virus, Aphid lethal paralysis virus, Lake Sinai virus, Solenopsis invicta virus, SINV) and 1 DNA virus, AmFV. The PCR reaction mixture contained 2×GoTaq reaction buffer (Promega, USA), 1 µM of the sense and antisense primers, and 1 µL of cDNA; the total reaction volume was increased to 20 µL using nuclease-free water. The PCR conditions were as follows: an initial denaturation step at 95 °C for 1 min; 33 cycles of 94 °C for 30 s, the suitable Tm for 30 s, and 72 °C for 1 min; and a final extension step at 72 °C for 10 min. The PCR products were separated using electrophoresis (1.2% agarose gel stained with GV II [Biomec, China]) and visualized using a FR-200A luminescent and fluorescent biological image analysis system (Furi, China). Product size was determined using a 100 bp molecular size marker. Because DWV-A/KV and AmFV have never been reported in France, we only looked for these two viruses in the Chinese samples.

We used multiplex PCR (as previously described [24]) to test the French samples for the presence of the six most common honey bee viruses: ABPV, IAPV, SBV, DWV, BQCV, and CBPV. The PCR reaction mixture contained 0.5 U of Diamond Taq^®^ polymerase (Invitrogen, Milan, Italy), 2.5 µL of 10× buffer (Invitrogen, Milan, Italy), 1.5 mM of MgCl_2_, 0.2 mM of each dNTP, 0.4 µM of the forward and reverse primers, and 100 ng of cDNA; the total reaction volume was increased to 25 µL using nuclease-free water. The PCR conditions were as follows: an initial denaturation step at 95 °C for 2 min; four cycles of 95 °C for 1 min, 54 °C for 1 min, and 72 °C for 1 min; 30 cycles of 95 °C for 1 min, 49 °C for 1 min, and 72 °C for 1 min; and a final extension step at 72 °C for 10 min. The PCR products were separated using electrophoresis (2.5% agarose gel stained with Red DNA Stain) and visualized using a UV light.

### 2.5. Sequencing and Phylogenetic Analyses

The PCR products were purified and sequenced by Sango Biotech (Shanghai, China) and by GATC Biotech (Konstanz, Germany). The sequences from the Chinese samples were assembled and manually corrected using Lasergene 8 and Geneious (v. 9) software.

For each virus, sequences were then aligned using the MAFFT algorithm [25] implemented in Geneious (v. 9) [26] and all the available homologous sequences found in the NCBI Nucleotide database. Maximum likelihood phylogenies were built using PhyML 3.0 online (http://www.atgc-montpellier.fr/phyml/) [27]. In each case, the best evolutionary model was determined using smart model selection (SMS) and the Akaike information criterion (AIC) [28]. Node support values were estimated using an approximate likelihood-ratio test (aLRT) [29].

The sequences of the viruses were submitted to GenBank under the accession numbers MF092814 to MF092832 for BQCV from China and MH133351 and MH133352 for BQCV from France; MF092819 to MF092825 for IAPV from China; MF092827 to MF092832 for DWV-A from China; MF092817 for AmFV from China; MF092826 for DWV-B from China; MF092818 for DWV-A/KV from China; and MH133361 for SBV from France. The DWV-A sequences VC-F1, VV-L2, VC-S2 were shorter than 200 bp long and were thus not attributed accession numbers.

## 3. Results

### 3.1. Virus Occurrence

We screened the vespid colonies for the presence of 11 viruses (Table 1). Several viruses were detected in all the vespid species sampled in China, with the exception of those from the Jiangxi and Qinghai provinces. Four honey bee viruses (CBPV, KBV, ALPV, and SINV) were not found in any of the vespids. IAPV and DWV-B were the most common viruses: they were present in nine individuals, representing five vespid species, and all the provinces sampled (Table 1). The third most common virus was AmFV, which was detected in six individuals, followed by BQCV and DWV-A/KV, which were detected in five individuals. LSV was found in pupae from the B2 colony. Interestingly, the honey bee viruses were most commonly found in pupae, not adults (Table 1). In the vespids sampled in France, the most common virus was DWV-A, followed by BQCV and SBV. Finally, ABPV was not observed in any vespids from either country.

In China, the co-occurrence of more than one virus in vespid colonies was fairly common for both adults and pupae (Table 2). In adults, all co-occurrences involved DWV. More co-occurrences were found in pupae than in adults, with up to six viruses detected in a single colony. The main viruses involved were AmFV, DWV, and IAPV. No co-occurrences were detected in French vespids.

### 3.2. Phylogenetic Relationships Among the Virus Sequences Found in Vespids

**Figure 2:**
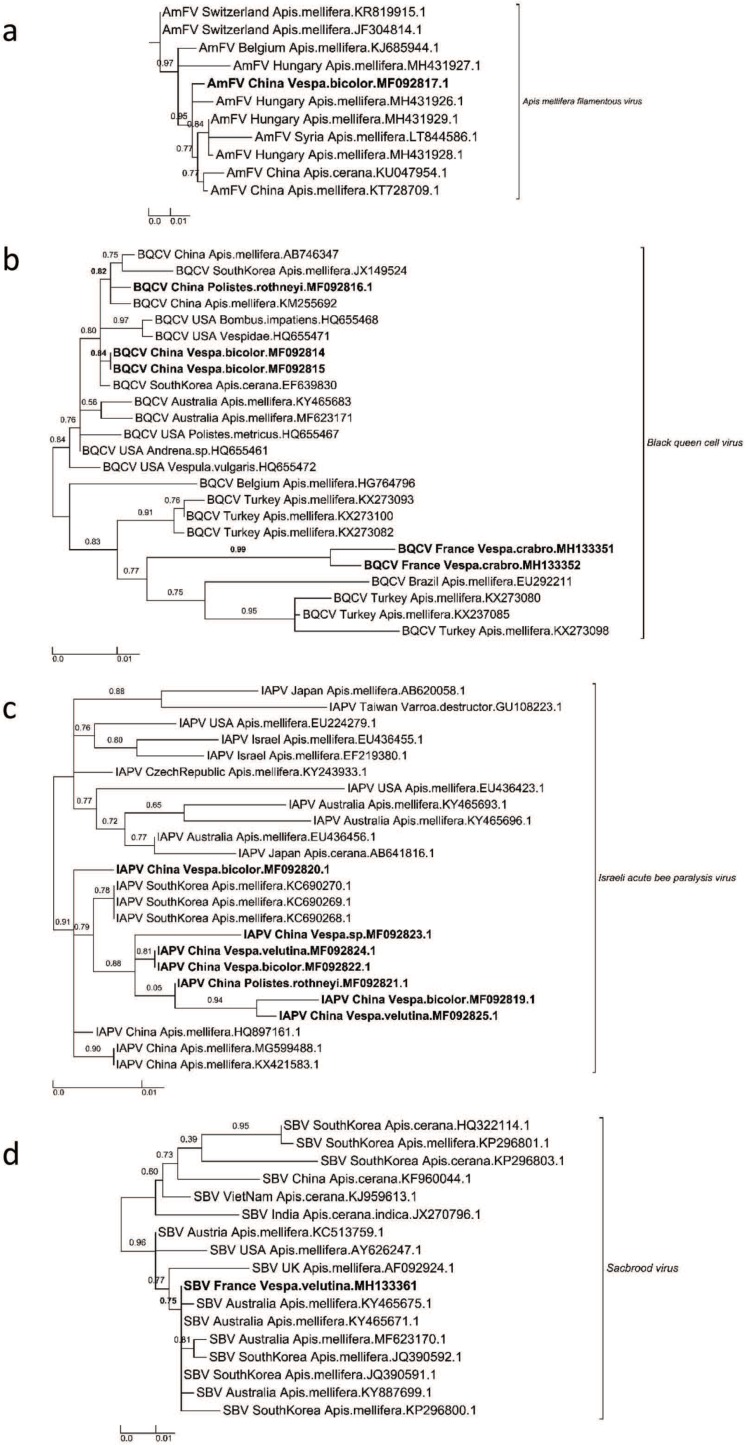
Maximum Likelihood phylogenetic trees of new AmFV, BQCV, IAPV, and SBV sequences found in China and France. (**a**) *Apis mellifera filamentous virus* phylogeny (524 sites, TN93+G model), (**b**) *Black queen cell virus* phylogeny (634 sites, GTR+I+G model), (**c**) Israeli acute bee paralysis virus (439 sites, TN93+I model) and (**d**) *Sacbrood virus* phylogeny (277 sites, GTR+G model). Names of countries and species for each known and new sequence were added in each leaf of the tree, joined with the corresponding GenBank accession number. The scale bars represent the substitution rate per site. The values correspond to the values of nodes supported by aLRT statistics.

Our phylogenetic analyses confirmed the species identity of the new AmFV sequence from China (B4/Beijing, *Polistes rothneyi*) (node support value = 0.95) (Figure 2a). The BQCV sequences from China and France formed different groups (Figure 2b). The French sequences clustered with European BQCV sequences (mainly from Turkey; node support value = 0.83), and the three Chinese BQCV sequences were found within a clade formed by Asian and US sequences (node support values = 0.84 and 0.82, respectively) (Figure 2b).

In the phylogeny for IAPV, six of the seven IAPV sequences from this study formed a subgroup (node support value = 0.88), and the sister group of this clade was the cluster of the IAPV sequences found in *A. mellifera* in South Korea (Figure 2c). The seventh sequence was most closely related to IAPV sequences in *A. mellifera* in China (node support value = 0.91). One SBV sequence was discovered in a hornet in France, and it was located within a clade formed by SBV sequences in *A. mellifera* in Australia and South Korea (node support value = 0.75) (Figure 2d).

**Figure 3:**
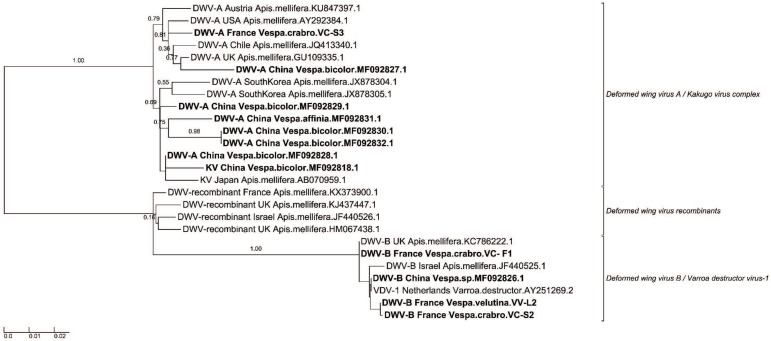
Concatenate Maximum Likelihood phylogenetic tree of *Deformed wing virus* sequences found in China and France. This phylogeny was built by concatenated four sequences’ alignment regions, due to multiple PCR detection primers used in protocols (Region 1: 2170–2438; region 2: 2843–3182; Region 3: 6111–6310; Region 4: 8377–8753) (1004 sites, GTR+G model). To tree topology of the concatenation was verified using each independent region in their respective phylogenetics tree (Supplementary Figure S2a–d). Country and species names for each known and new sequence were added in each leaf of the tree, joined with the corresponding GenBank accession number. The scale bars represent the substitution rate per site. The values correspond to the values of nodes supporting by aLRT statistics.

The phylogeny shows that, as expected, the DWV complex comprises two genotypes, A and B (formerly *V. destructor* virus-1). Interestingly, both DWV genotypes were represented in the sequences obtained from vespids in China and France. Because different DWV loci were amplified for the Chinese and French samples, separate phylogenetic trees were reconstructed for each country (Figure S3), and we generated a combined phylogenetic tree using concatenated aligned sequences (Figure 3). Both approaches gave similar results.

## 4. Discussion

Vespids are widely distributed across the world, from tropical to temperate regions. While some species can help disperse plant seeds, such as those of *Stemona tuberosa* [30], other species are predators. For example, the yellow-legged hornet (*V. velutina*) is a specialized predator of honey bee foragers and thus represents a serious threat to the apiculture industry and pollination ecosystem services [31]. This species was introduced into Europe in 2004 and since then it has placed significant biological stress on honey bee colonies.

Our study aimed to take a first look at the occurrence of 11 honey bee viruses in vespid species collected in China and France; it also examined phylogenetic relationships among virus sequences. The results show that several viruses were commonly present and broadly geographically distributed in a variety of vespid species. In addition, we found more viruses in pupae than in adults, which is not surprising given that adults feed larvae large quantities of masticated bees. While we did not investigate infection dynamics, this finding might suggest that pupae are more suitable or more susceptible hosts than adults, if the viruses are capable of replicating in vespids. In addition, we also did not detect the pupae from France vespids due to limited samples. Such stage-dependent susceptibility has been described for several viruses that infect both *A. mellifera* and *A. cerana* [32,33]. Further experimental research is now needed to test whether (1) honey bee viruses can replicate in hornets (2) hornets can then transmit the viruses to honey bees and (3) pupae from France vespids carry more honey bee viruses than adults.

Based on our phylogenetic analyses, the viruses infecting vespids do not form specific clades. This result could suggest that the honey bee viruses are not transmitted among vespids but rather from honey bees to vespids. It might also indicate that, if replication occurs in vespids, it primarily results from recent spillovers from honey bees. A recent study showed that such spillovers could result in DWV-B and KBV infections in the yellow-legged hornet, including a case in which an individual was symptomatic (i.e., had deformed wings) [34]. In our study, DWV was the most common virus in both China and France, which is unsurprising given its frequency in honey bees worldwide [4]. Both genotypes—DWV-A and DWV-B—were observed. Future research might reveal more specific replication dynamics in vespids.

IAPV was first identified in Israel in 2004 but has since been found to have a worldwide distribution [35]. It is a virus that displays high genetic diversity [36]. IAPV is quite common in China [37], and its replicative form has previously been detected in the yellow-legged hornet [18]. Consequently, there may be interspecific transmission of IAPV, an issue that should be explored further in epidemiological studies. In contrast, BQCV sequences from vespids in China and France formed two different groups. The long branches in the phylogeny suggest that this virus displays pronounced genetic diversity. As a result, concerted efforts should be made to construct a more global phylogeny for BQCV to better identify its possible adaptive diversity and host associations.

Although AmFV was first identified in the honey bee in 1963 [38] and its complete dsDNA genome was recently published [39], we actually know little about the virus’s occurrence [40], especially in China. It is thus quite notable that we observed AmFV in several hornets and especially in pupae. There could be several explanations for this result. First, AmFV might be more common in China than in Europe, where it was first detected. Second, honey bees might be frequently infected with AmFV, but we could be unaware of this fact because there is no specific monitoring of this virus. If AmFV prevalence is high in honey bees, vespids—and notably vespid larvae/pupae—may be more likely to ingest and accumulate the virus, which might explain why AmFV is so common in vespids in China. While AmFV could also be infectious in vespids, our phylogenetic analyses revealed no evidence that AmFV sequences from vespids were distinct from AmFV sequences from honey bees, which could suggest that vespids do not carry a specific form of the virus. Complete genome sequencing might reveal more divergent molecular markers that would allow us to better discriminate among virus sequences. Overall, our findings for AmFV highlight the need to further investigate the dynamics of this virus in honey bees and vespids.

## 5. Conclusions

To summarize, we characterized the occurrence of the 11 most important honey bee viruses in vespid species in China and France. We also identified differences in virus co-occurrence among different vespid species in China. All the virus sequences that we obtained from vespids were closely related to sequences obtained from honey bees, likely indicating that the vespids acquired the viruses while feeding on bees. However, further research is needed to explore infection dynamics in vespids, and the potential for virus transmission between vespids and honey bees.

## Figures and Tables

**Table 1:** Presence of major honey bee viruses in various vespid species.

Country	Developmental Stage	Colony ID/Province (Species)	AmFV	IAPV	DWV-A	DWV-B	DWV-A/KV	CSBV	SBV	BQCV	LSV
China	Adult	B1/Beijing (*Vespa bicolor*)	nt	nt	+	nt	+	nt	nt	nt	nt
	Pupa		+	+	+	nt	+	nt	nt	nt	nt
	Adult	B2/Beijing (*Vespa bicolor*)	nt	nt	+	nt	nt	nt	nt	+	nt
	Pupa		+	+	+	nt	+	+	nt	+	+
	Adult	B3/Beijing (*Vespa bicolor*)	nt	nt	nt	nt	nt	nt	nt	nt	nt
	Pupa		nt	nt	nt	nt	nt	nt	nt	nt	nt
	Adult	B4/Beijing (*Polistes rothneyi*)	+	nt	nt	nt	nt	nt	nt	nt	nt
	Pupa		+	nt	nt	nt	nt	nt	nt	+	nt
	Adult	B5/Beijing (*Polistes rothneyi*)	nt	nt	nt	nt	nt	nt	nt	nt	nt
	Pupa		+	+	nt	nt	nt	nt	nt	nt	nt
	Adult	Q1/Qinghai *(Vespa koreansis)*	nt	nt	nt	nt	nt	nt	nt	nt	nt
	Adult	GD1/Guangdong (*Vespa affinia*)	nt	nt	+	nt	nt	nt	nt	nt	nt
	Pupa		nt	+	+	nt	nt	nt	nt	nt	nt
	Adult	GD2/Guangdong (*Vespa velutina*)	nt	nt	nt	nt	nt	nt	nt	nt	nt
	Pupa		nt	+	nt	nt	nt	nt	nt	nt	nt
	Adult	GD3/Guangdong (*Vespa velutina*)	nt	nt	nt	nt	nt	nt	nt	nt	nt
	Pupa		nt	+	nt	nt	nt	nt	nt	nt	nt
	Adult	GD4/Guangdong (*Vespa sp*.)	nt	nt	nt	nt	nt	nt	nt	nt	nt
	Pupa		nt	+	+	+	+	nt	nt	+	nt
	Adult	GD5/Guangdong (*Vespa bicolor*)	nt	+	+	nt	nt	nt	nt	nt	nt
	Pupa		nt	+	+	nt	nt	nt	nt	nt	nt
	Adult	JX/Jiangxi *(Vespa bicolor)*	nt	nt	nt	nt	nt	nt	nt	nt	nt
	Pupa		nt	nt	nt	nt	nt	nt	nt	nt	nt
France	Adult	Fondettes (*Vespa crabro*)	nt	nt	+	nt	nt	nt	nt	nt	nt
	Adult	Saché (*Vespa crabro*)	nt	nt	+	nt	nt	nt	nt	nt	nt
	Adult	Saché (*Vespa crabro*)	nt	nt	+	nt	nt	nt	nt	nt	nt
	Adult	Lussault-sur-Loire (*Vespa velutina*)	nt	nt	+	nt	nt	nt	nt	nt	nt
	Adult	Lussault-sur-Loire (*Vespa velutina*)	nt	nt	nt	nt	nt	nt	+	nt	nt
	Adult	Fondettes (*Vespa crabro*)	nt	nt	nt	nt	nt	nt	nt	+	nt
	Adult	Tours (*Vespa crabro*)	nt	nt	nt	nt	nt	nt	nt	+	nt

nt: not tested; only the positive samples are included in the table.

**Table 2:** Co-occurrence of virus species in vespid colonies in China.

Colony ID	Vespid Species	Developmental Stage	Viruses Present
B2	*Vespa bicolor*	Adult	DWV+BQCV
GD5	*Vespa bicolor*	Adult	IAPV+DWV
B4	*Polistes rothneyi*	Pupa	AmFV+BQCV
B5	*Polistes rothneyi*	Pupa	AmFV+IAPV
GD1	*Vespa affinia*	Pupa	DWV+IAPV
B1	*Vespa bicolor*	Pupa	AmFV+IAPV+DWV
GD4	*Vespa sp*.	Pupa	IAPV+DWV+BQCV
B2	*Vespa bicolor*	Pupa	AmFV+IAPV+DWV+CSBV+BQCV+LSV

## Supplementary Materials

The supplementary materials are available online at https://www.mdpi.com/1999-4915/12/1/6/s1. They include Table S1. PCR primers used to identify the honey bee viruses in vespids collected in China and France. Figure S1. Picture of a yellow-legged hornet (*Vespa velutina*) attacking a honey bee. Figure S2. The quality of the RNA extracted from the vespid samples. M, DNA ladder; the numbers 1–10 refer to the colony IDs B1, B3, B4, B5, Q1, GD1, GD2, GD3, GD4, and JX, respectively. Figure S3. Separate maximum likelihood phylogenetic trees for each *Deformed wing virus* (DWV) sequence region in China and France. (a) Region 1: 2170–2438 (201 sites, HKY85+I model); (b) Region 2: 2843–3182 (340 sites, HKY85+I model); (c) Region 3: 6111–6310 (152 sites, TN93+I model); and (d) Region 4: 8377–8753 (311 sites, HKY85+G model). The country names, species names, and GenBank accession numbers were also added to each branch of the tree. The scale bars represent the substitution rate per site, and the values above the nodes are the aLRT statistics.
