# Supplementary material for: Occurrence and Molecular Phylogeny of Honey Bee Viruses in Vespids
Source: Viruses. 2019 Dec 19;12(1):6. doi: 10.3390/v12010006 (PMC7019919; doi:10.3390/v12010006)
Supplement: Supplementary file 1 [file viruses-12-00006-s001.pdf]

Table S1. Primers used for detection honey bee viruses in current study.

**Virus/Pathogen**	**Primer**	**Sequence**	**Product Length (bp)**	**Reference**
IAPV	IAPVF	AGA CAC CAA TCA CGG ACC TCA C	474	1
	IAPVR	AGA TTT GTC TGT CTC CCA GTG CAC		
SBV	SBVF	ATA TAC GGT GCG AGA ACT GC	879	2
	SBVR	CTC GGT AAT AAC GCC ACT GT		
CSBV	CSBVF	CCTGGGAAGTTTGCTAGTATTTACG	161	6
	CSBVR	CCTATCACATCCATCTGGGTCAG		
DWV-A	DWVF	GACTGAACCAAATCCGATGTC	376	3
	DWVR	TCTCAAGTTCGGGACGCATTC		
DWV-B	VDV-1F	TGGCTAATCGACGTAAAGCA	200	5
	VDV-1R	ACTAATCTCTGAGCCAACACGT		
DWV-A/KV	KVF	GATATGACTGTATCCTCCATAGCATCTC	396	4
	KVR	GTATGAAACATATGGCACCTCAAAAGTA		
CBPV	CBPVF	TCA GAC ACC GAA TCT GAT TAT TG	570	7
	CBPVR	ACT ACT AGA AAC TCG TCG CTT CG		
ABPV	ABPVF	TTA TGT GTC CAG AGA CTG TAT	900	8
	ABPVR	GCT CCT ATT GCT CGG TTT TTC		
BQCV	BQCVF	TGG TCA GCT CCC ACT ACC TTA AAC	700	9
	BQCVR	GCA ACA AGA AGA AAC GTA AAC CAC		
LSV	LSVF	tgtaaaacgacggccagtGCCWCGRYTGYTRGTDCCYCC	577	15
	LSVR	caggaaacagctatgaccGAVGTGGNGGNGCNAGATARAGT		
SINV	SINVF	CAATAGGCACCAACGTATATAGTAGAGATTGGA	253	10
	SINVR	GGAATGGGTCATCATATAGAAGAATTG		
ALPV	ALPVF	GCGTACCATACTACTCACCATATTTATTTA	140	11
	ALPVR	AGTTAATCCATAAAGTGCAATCTACAATAC		
KBV	KBVF	TATGCTGAACAACGCAAAGA	639	12
	KBVR	ACAACACGATGTCTGGGTTT		
AmFV	AmFV-F	CAGAGAATTCGGTTTTTGTGAGTG	550	13
	AmFV-R	CATGGTGGCCAAGTCTTGCT		
**Primers Used for French Samples**				
IAPV	AIVf	GGTGCCCTATTTAGGGTGAGGA	158	14
	IAPVr	GGGAGTATTGCTTTCTTGTTGTG		
DWV	DWVf	TGGTCAATTACAAGCTACTTGG	269	14
	DWVr	TAGTTGGACCAGTAGCACTCAT		
SBV	SBVf	CGTAATTGCGGAGTGGAAAGATT	342	14
	SBVr	AGATTCCTTCGAGGGTACCTCATC		
ABPV	AIVf	GGTGCCCTATTTAGGGTGAGGA	460	14
	ABPVr	ACTACAGAAGGCAATGTCCAAGA		
BQCV	BQCVf	CTTTATCGAGGAGGAGTTCGAGT	536	14
	BQCVr	GCAATAGATAAAGTGAGCCCTCC		
CBPV	CBPVf	AACCTGCCTCAACACAGGCAAC	774	14
	CBPVr	ACATCTCTTCTTCGGTGTCAGCC		
LSV	LSV-F-1791	tgtaaaacgacggccagtGCCWCGRYTGYTRGTDCCYCC	616	15
	LSV-R-2368	caggaaacagctatgaccGAVGTGGNGGNGCNAGATARAGT		
Universal sequencing primers	M13-FP	tgtaaaacgacggccagt		15
	M13-RP	caggaaacagctatgacc		

References

1. Maori, E.; Tanne, E.; Sela, I. Reciprocal sequence exchange between non-retro viruses and hosts leading to the appearance of new host phenotypes. *Virology* **2007**, *362*, 342–349.
2. Grabensteiner, E.; Ritter, W.; Carter, M.J.; Davison, S.; Pechhacker, H.; Kolodziejek, J.; Boecking, O.; Derakhshifar, I.; Moosbeckhofer, R.; Licek, E.; Nowotny, N. Sacbrood virus of the honeybee (Apis mellifera): Rapid identification and phylogenetic analysis using reverse transcription-PCR. *Clin. Diagn. Lab. Immun.* **2001**, *8*, 38–104.
3. Chen, Y.P.; Higgins, J.A.; Feldlaufer, M.F. Quantitative analysis of deformed wing virus infection in the

honey bee, Apis mellifera L. by real-time RT-PCR. *Appli. Environ. Microbiol.* **2004**, *71*, 436–441.

4. Fujiyuki, T.; Ohka, S.; Takeuchi, H.; et al. Prevalence and Phylogeny of Kakugo Virus, a Novel Insect Picorna-Like Virus That Infects the Honeybee (Apis mellifera L.), under Various Colony Conditions. *J. Virol.* **2006**, *80*, 11528.
5. Zioni, N.; Soroker, V.; Chejanovsky, N. Replication of Varroa destructor virus 1 (VDV-1) and a Varroa destructor virus 1–deformed wing virus recombinant (VDV-1–DWV) in the head of the honey bee. *Virology* **2011**, *417*, 106–112.
6. Ma, M.X. et al. Genetic characterization of VP1 gene of seven Sacbrood virus isolated from three provinces in northern China during the years 2008–2012. *Virus Res.* **2013**, *176*, 78–82, doi:10.1016/j.virusres.2013.04.018 (2013).
7. Berényi, O.; Bakonyi, T.; Derakhshifar, I.; Köglberger, H.; Nowotny, N. Occurrence of six honeybee viruses in diseased Austrian apiaries. *Appl. Environ. Microbiol.* **2006**, *72*, 2414–2420.
8. Blanchard, P.; Ribière, M.; Celle, O.; Lallemand, P.; Schurr, F.; Olivier, V.; Iscache, A.L.; Faucon, J.P. Evaluation of a real-time two-step RT-PCR assay for quantitation of Chronic bee paralysis virus (CBPV) genome in experimentally-infected bee tissues and in life. *Appl. Environ. Microbiol.* **2007**, *73*, 7711–7716.
9. Benjeddou, M.; Leat, N.; Allsopp, M.; Davison, S. Detection of acute bee paralysis virus and black queen cell virus from honeybees by reverse transcriptase PCR. Appl. *Environ. Microbiol.* **2001**, *67*, 2384–2387.
10. Hashimoto, Y; Valles, S.M.; Strong C.A. Detection and quantitation of Solenopsis invicta virus in fire ants by real-time PCR. *J. Virol. Methods* **2007**, *140*, 132–139.
11. Dombrovsky, A.; Luria, N. TheNerium oleanderaphidAphis neriiis tolerant to a local isolate ofAphid lethal paralysis virus(ALPV). *Virus Genes* **2013**, *46*, 354–361.
12. Stolz, D.; Shen, X.R.; Boggis, C.; Sisson, G. Molecular diagnosis of Kashmir bee virus infection, *J. Apic. Res.* **1995**, *34*, 153–160.
13. Hartmann, U.; Forsgren, E.; Charrière, J.-D.; Neumann, P.; Gauthier, L. Dynamics of Apis melliferaFilamentous Virus (AmFV) Infections in Honey Bees and Relationships with Other Parasites. *Viruses* **2015**, *7*, 2654–2667.
14. Sguazza, G.H.; Reynaldi, F.J.; Galosi, C.M.; Pecoraro, M.R. Simultaneous detection of bee viruses by multiplex PCR. *J. Virol Methods.* **2013**, *194*, 102–106.
15. Bigot, D.; Dalmon, A.; Roy, B.; Hou, C.; Germain, M.; Romary, M.; et al. The discovery of Halictivirus resolves the Sinaivirus phylogeny. *J. Gen. Virol.* **2017**, *98*, 2864–2875.

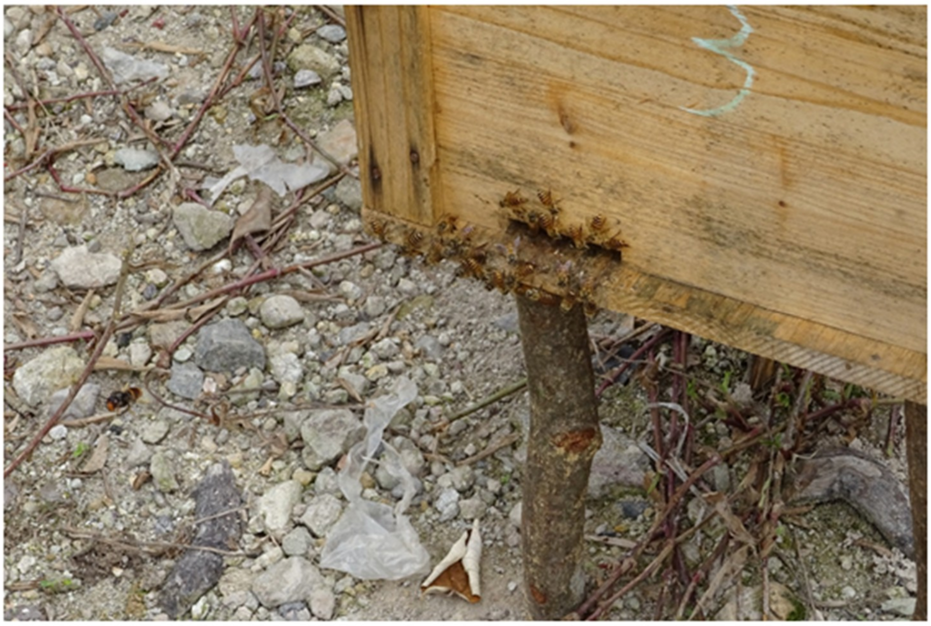

Figure S1. Picture of a yellow-legged hornet (*Vespa velutina*) attacking a honey bee.

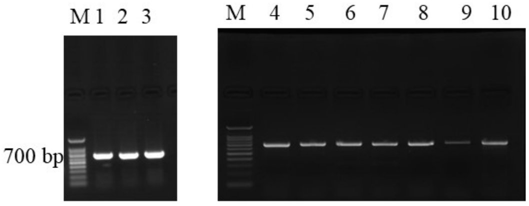

Figure S2. The quality of the RNA extracted from the vespid samples. M, DNA ladder; the numbers 1–10 refer to the colony IDs B1, B3, B4, B5, Q1, GD1, GD2, GD3, GD4, and JX, respectively.

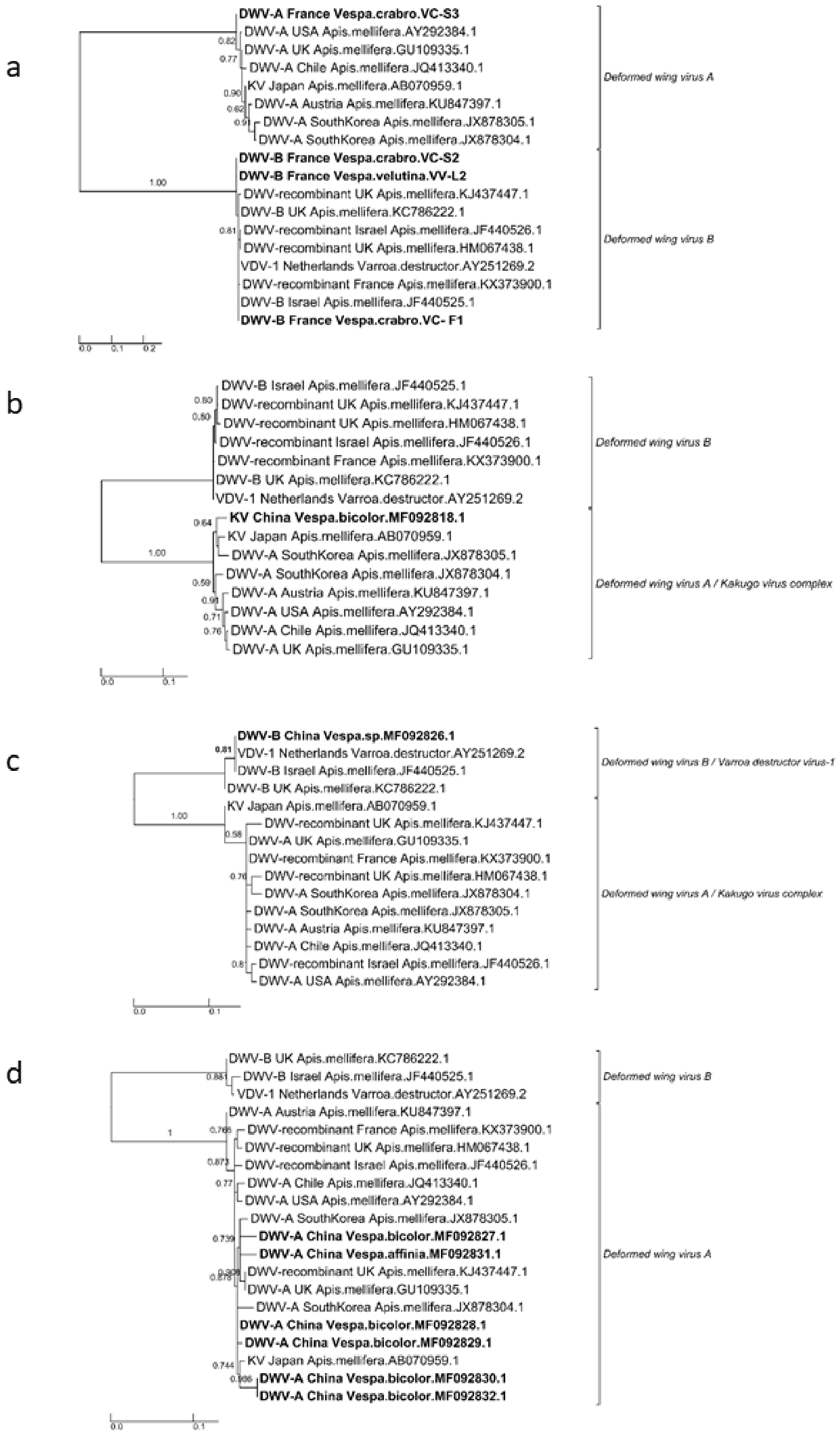

Figure S3. Separate maximum likelihood phylogenetic trees for each *Deformed wing virus* (DWV) sequence region in China and France. (a) Region 1: 2170–2438 (201 sites, HKY85+I model); (b) Region 2: 2843–3182 (340 sites, HKY85+I model); (c) Region 3: 6111–6310 (152 sites, TN93+I model); and (d) Region 4: 8377–8753 (311 sites, HKY85+G model). The country names, species names, and GenBank accession numbers were also added to each branch of the tree. The scale bars represent the substitution rate per site, and the values above the nodes are the aLRT statistics.
